# Potential application of *Pseudomonas stutzeri* W228 for removal of copper and lead from marine environments

**DOI:** 10.1371/journal.pone.0240486

**Published:** 2020-10-26

**Authors:** Carolina Coelho da Costa Waite, Guilherme Oliveira Andrade da Silva, José Augusto Pires Bitencourt, Luciana Pereira Torres Chequer, Simone Pennafirme, Diogo de Azevedo Jurelevicius, Lucy Seldin, Mirian Araújo Carlos Crapez

**Affiliations:** 1 Programa de Biologia Marinha e Ambientes Costeiros, Instituto de Biologia, Universidade Federal Fluminense, Niterói, RJ, Brazil; 2 Instituto Tecnológico Vale de Desenvolvimento Sustentável, Nazaré, Belém, PA, Brazil; 3 Instituto de Microbiologia Professor Paulo de Góes, Centro de Ciências da Saúde.CCS—Ilha do Fundão, Universidade Federal do Rio de Janeiro, Rio de Janeiro, RJ, Brazil; King Saud University, SAUDI ARABIA

## Abstract

High concentrations of metals in the environment alter bacterial diversity, selecting resistant and tolerant species. The study evaluated the selection of a potential bacterial strain from Sepetiba Bay-Rio de Janeiro, Brazil marine sediments to remove Cu and Pb. The bacterial strain isolated from the sediments was used in three different bioassays: (1) Cu at concentrations of 0 (control), 6 and 50 μg.mL^-1^; (2) Pb at concentrations of 0 (control), 6 and 50 μg.mL^-1^; (3) Cu + Pb in concentrations of 3 μg.mL^-1^ Cu + 3 μg.mL^-1^ Pb (6 μg.mL^-1^) and 25 μg.mL^-1^ Cu + 25 μg.mL^-1^ Pb (50 μg.mL^-1^). The number of cells and the enzymatic activities of dehydrogenases and esterases were quantified. Results of taxonomic identification indicated the selection of the *Pseudomonas stutzeri* W228 strain, showing a greater degree of similarity (±73%) with the database used. There was no significant variation in the number of cells, 10^8^ cells.mL^-1^, which represents a high biomass production in the presence of stressors. However, we observed a reduction in dehydrogenase activity at all tested concentrations of Cu, Pb and Cu + Pb. The activity of esterase increased, indicating a higher energy demand to complete the bacterial life cycle. The study showed significant results for the absorption of Pb by the extracellular polymeric substances (EPS) and the efflux of Cu. The capacity of Pb absorption by EPS can be considered a resistance mechanism, as well as the efflux of Cu, so that the available EPS sites could be occupied by the most toxic ions demonstrating that *Pseudomonas stutzeri* is resistant to Pb and Cu.

## 1. Introduction

Metals are a serious threat to ecosystems, due to toxicity, non-degradability, persistence, with the possibility of bioaccumulating in the food web [1]. Soils/sediments transport and store potentially dangerous metals through natural emissions from volcanoes, transport of continental dust, and weathering of rocks [2].

Microorganisms are the active agents in all biogeochemical cycles, including metals, because they interact with the latter, changing speciation and mobility. They mobilize metals through autotrophic and heterotrophic metabolisms, enabling bioleaching [3, 4]. Microorganisms also promote the immobilization of metals through intracellular accumulation or binding to EPS (extracellular polymeric substance) [5, 6].

In high concentrations, Cu is toxic to microorganisms because it can promote protein denaturation and membrane damage by thiol-binding, replaces calcium and zinc, favors the formation of free radicals leading to reduced defense against lipid peroxidation [7, 8]. Pb is disposed of in the environment as Pb oxides and carbonates, Pb-metal-oxyanion complexes, and it is considered a priority pollutant because some forms of lead-salt, such as lead acetate or nitrate induce mutagenicity and DNA breaks [9]. *Pseudomonas stutzeri* has particular metabolic properties such as: denitrification; which makes them relevant to the study of gene transfer in the environment; several strains are capable of fixing dinitrogen; and others participate in the degradation of pollutants or interact with toxic metals [10].

To tackle this persistent environmental concern, novel technologies involving multidisciplinary approaches and utilizing microorganisms with enhanced bioremediation capabilities have been proposed [11, 12]. Microbial bioremediation is an environmentally friendly approach to treat noxious conditions in the current scenario of ongoing and increasing pollution. It is an attractive approach compared to conventional methods because it does not alter the natural microenvironment and maintains ecosystem balance [13].

This work aimed to isolate copper-resistant bacteria in liquid culture medium containing sediment sampled in an environment with a history of contamination and then to verify the resistance to Pb in bioassays, providing information on the concentrations of Cu, Pb and Cu + Pb that can be used by *P*. *stutzeri* strain W 228 **Lehman and Neumann, 1896–1927** in bioremediation technology.

## 2. Material and methods

### 2.1. Sampling location

**Fig 1:**
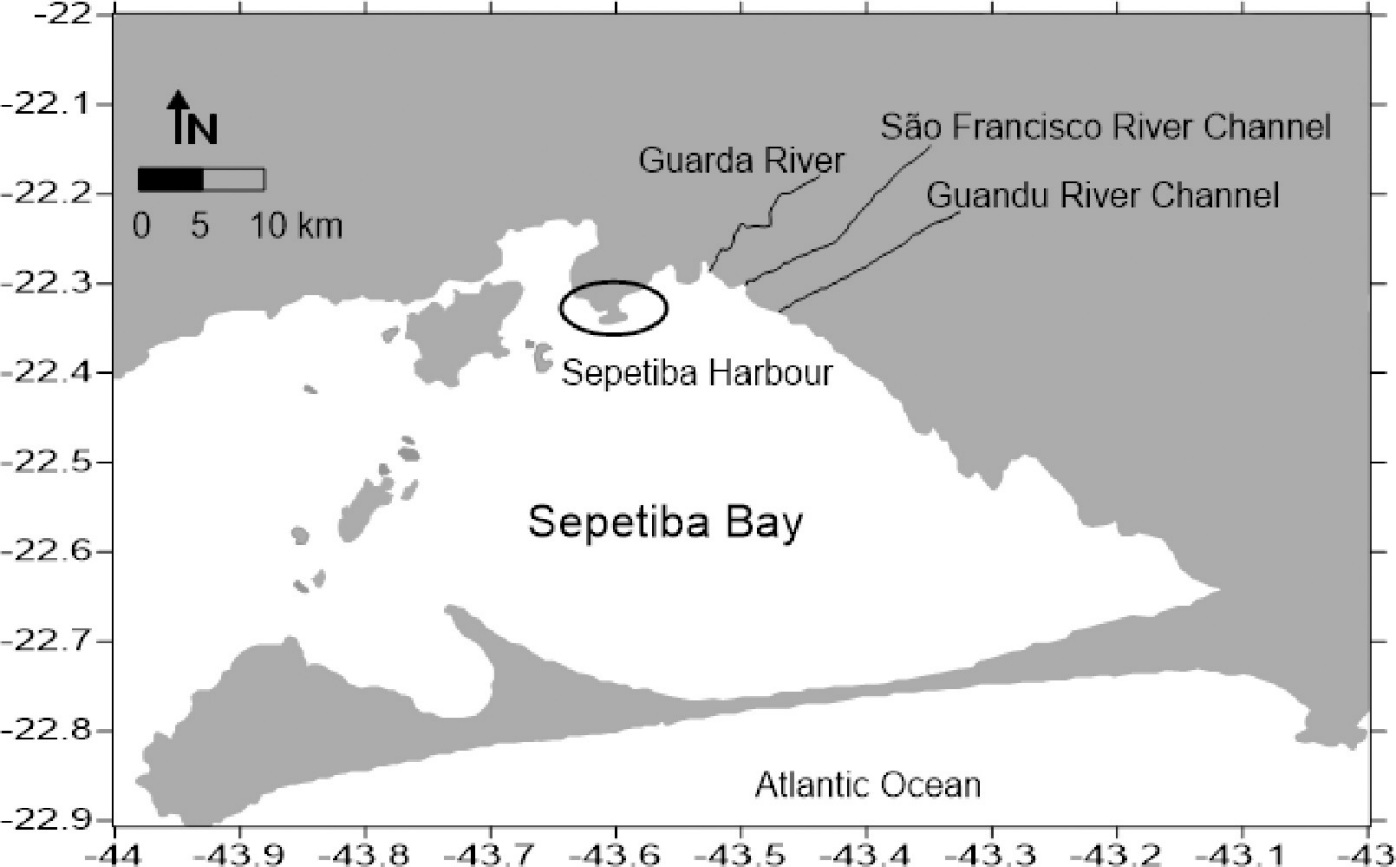
Map of the sampling point (in detail), Saco do Engenho—Sepetiba Bay, RJ, Brazil.

Sepetiba Bay (Fig 1) is located approximately at latitude 23°S and longitude 44°W, about 60 km south of Rio de Janeiro city, Brazil. It is a semi-enclosed bay with an area of 447 km^2^, an average depth of 6.0 m, and a tidal variation of less than 2.0 m. The water replenishment time is about 100 hours [14]. It suffers high pressure of anthropic factors due expansion of industrial activities [15]. According to Pinto et al. [16], the sediments have 0.5 mg.kg^-1^ of Cd, 14.4 mg.kg^-1^ Cu, 23.4 mg.kg^-1^ Ni, 24.2 mg.kg^-1^ Pb and 82.6 mg.kg^-1^ Zn. The bacterial consortia were isolated from superficial sediment samples from Saco do Engenho tidal channel, Sepetiba Bay, Rio de Janeiro, Brazil (43°48'25.01''W; 22°54'56.38''E) and belongs to the collection of the Microbial Ecology Laboratory at Universidade Federal Fluminense (Fig 1). None legal permission was required for sampling at this site because only sediment was sampled for research purposes.

### 2.2. Isolation of bacterial consortia and bioassay

The process of bacterial consortia isolation was carried out according to Fonseca et al. [15] and Baptista-Neto et al. [17]. In the initial step, a fresh sediment sample from Sepetiba Bay was placed into a liquid sterile medium in a proportion of 1:10. The incubation occurred for ten days at 37°C, with a pH of around 6 (buffered solution) until we attained 10^8^ cells.mL^−1^. The medium used was made accordingly to Madigan et al. [18], with a diluted (1: 1 v / v) and filtered seawater (Millipore, Cellulose, 0.45 μm), 5 g.L^-1^ of yeast extract (fount of vital nutrients and trace elements) and 5 g.L^-1^ of urea as the primary source of nitrogen, in Erlenmeyer flasks [5]. Then, an aliquot of this culture (1:10) was inoculated into a new sterile liquid culture medium and maintained up to 10 days of incubation at room temperature (25°C). To acclimate the bacterial consortia at room temperature and ensure that they would grow in the absence of the sediment. In the last step, It sought isolation of bacterial consortia resistant to metal. 5 mL of the acclimatization step was placed into a new sterile culture medium with 50 μg Cu.mL^−1^, and incubated it for 10 days to attain a cell density of 10^8^ cells.mL^−1^. This bacterial culture selected was maintained in the laboratory in the same type of liquid medium without Cu.

The bioassays were conducted in Erlenmeyer flasks (125 mL) at 37°C, each of which contained 5 mL of the isolated bacterial consortia resistant to Cu and 50 mL of sterile culture medium [18]. Cu and Pb solutions of 6 and 50 μg.mL^-1^, and the concentration of Cu + Pb was 3 and 25 μg.mL^-1^ of each metal, prepared from their respective stocks sterile solutions 1000 μg.mL^-1^. The reagents were analytical purity (Sigma Germany). Cu in low concentrations is used by the cell as a micronutrient. However, in high concentrations it can be toxic to the cells of any living being, as it competes with other metals for protein binding sites [19]. The lowest concentration of 6 μg.mL^-1^ is the maximum allowed content of Pb (the most toxic metal tested) in human blood, according to Brazilian laws [20]. The maximum concentration of 50 μg.mL^-1^ is the Pb content found in the sediments of Sepetiba Bay, according to Abuchacra et al. [21].

### 2.3. Classification of bacterial consortia by denaturing gradient gel electrophoresis (DGGE) and analysis of the 16S rRNA gene sequence library

PCR was performed using the primers U968F (5'-GAACGCGAAGAACCTTAC-3) and L1401R (5'-CGGTGTGTACAAGACCC-3') [22]. Amplification conditions were as follows: initial denaturation of double-stranded DNA for 3 min at 94°C; followed by 35 cycles consisting of 1 min at 94°C, 1 min and 30 s at 48°C and 1 min at 72°C; and extension for 10 min at 72°C [23]. DGGE was carried out using the INGENYphorU system (INGENY®, Leiden, NL). Aliquots of the PCR products (10 μL) were mixed with 10 μL of the running dye and this mixture was applied to an 8% (w/v) polyacrylamide gel containing a denaturing concentration gradient of 46.5–60% (urea and formamide), and electrophoresed as described by Heuer et al. [24]. Bands extracted from the DGGE gel were purified by PCR Clean-Up System (Promega®) and reamplified by PCR using the same conditions described above. Purified amplicons were then cloned using the InsTAclone® PCR cloning kit (Fermentas, Maryland, USA) following the manufacturer's instructions. Transconjugant colonies were selected by the α-complementation system of the β-galactosidase gene [25]. We established presence of the insert in the plasmid of the transconjugants by PCR using the primer pair M13F and M13R, [26]. The amplification reactions were performed with an initial denaturation cycle at 97°C for 3 min, followed by 40 cycles of 94°C for 30 s, 60°C for 30 s, and 72°C for 90 s, with a final extension at 72°C for 5 min. Plasmids from the obtained transconjugants were then sequenced using the primer M13F on an ABI Prism 3100 automated sequencer (Applied Biosystems Inc., CA, USA) by Macrogen (South Korea).

Plasmid vector region were manually removed and sequences were analyzed using the VecScreen® tool (National Center for Biotechnology Information, NCBI http://www.ncbi.nlm.nih.gov). The sequences were deposited on NCBI Genbank database with code MT547374 to MT547397.

### 2.4. Cell number quantification

Aliquots of the culture medium (2 mL) were filtered through a sterile Millipore membrane (pore diameter of 0.22 μm) [27] and stained with acridine orange fluorochrome. Cells were enumerated under epifluorescent microscopy at 1000× magnification (Axioskop 1, Zeiss, triple filter Texas Red–DAPI–fluorescein isothiocyanate), in accordance with Kepner and Pratt [28].

### 2.5. Dehydrogenase activity quantification

The use of iodonitrotetrazolium (INT) is the most accepted method for measuring redox reaction in cells. [29, 30]. Dehydrogenase activity was evaluated according to Stubberfield and Shaw [31]. For each bioassay, 1 mL aliquots in triplicate of each culture medium receive 0.2 mL of 8 mM INT. A control was prepared by replacing the INT solution with distilled water. After vortexing, the tubes were incubated in the dark. After 35 minutes, 5mL of methanol was added in each tube. The extractable solution was centrifuged (5 min at 2500 rpm), and its absorbance was determined at 458 nm with a spectrophotometer. Dehydrogenase activity expressed in μL INT-F / m was calculated by comparing absorbance values for a standard curve of 0.15 mM INT-Formazan methanol.

### 2.6. Quantification of esterase activity

Fluorescence diacetate (FDA) is used to quantify the hydrolysis of ester bonds from biopolymers greater than 600 Da [32]. Quantification of esterase enzyme (EST) activity was performed according to Stubberfield & Shaw [31]. The method is based on the estimate of the fluorescein produced in the sample treated with fluorescein diacetate solution and incubated 24°C for 75 minutes. The relationship between fluorescein concentration and optical density is always linear, the results are expressed in μg fluorescein / mL. The reading was made using an optical spectrophotometer (Spectronic 20D) at a wavelength of 490 nm.

### 2.7. Metal quantification

Metal concentrations were determined using the supernatants of the culture media filtered in the initial and final stages of the bioassay from the dilutions of the samples in 0.1 M HCl. The metals were quantified by atomic absorption spectrometry (Atomic absorption spectrometer Fast Sequential Varian AA 240FS detection sensitivity for Cu and Pb of 0.005 ppm and 0.09 ppm, respectively) at the Eduardo Penna Franca Radioisotope Laboratory in the Carlos Chagas Filho Biophysics Institute, UFRJ. To test the efficiency of our analytical approach, we use the same stock solutions used to prepare the bioassays, with a> 90% recovery for Cu and Pb.

### 2.8. Data analysis

Analyses were performed in triplicate, and mean values were calculated for each sample. We performed statistical analyses in the free software R-Project version 3.0.2. [33], using the ggplot2 package [34]. Data were standardized using the procedure "varying for variables with arbitrary zero" proposed by Milligan and Cooper [35]. Kruskal-Wallis was used to verify the differences between concentrations and time of exposure to metals of each bioassay using the R: language and environment statistical computing (p ≤ 0.05). All analyses were performed in triplicate. Prior to multivariate analysis, data were tested for normality (Shapiro-Wilk test) and homogeneity (Levene test). Principal component analysis (PCA) was applied to test the hypothesis that more than one independent variable had an effect on a set of dependent variables, eliminating most of the systemic variation of all the independent variables tested [36].

## 3. Results

**Fig 2:**
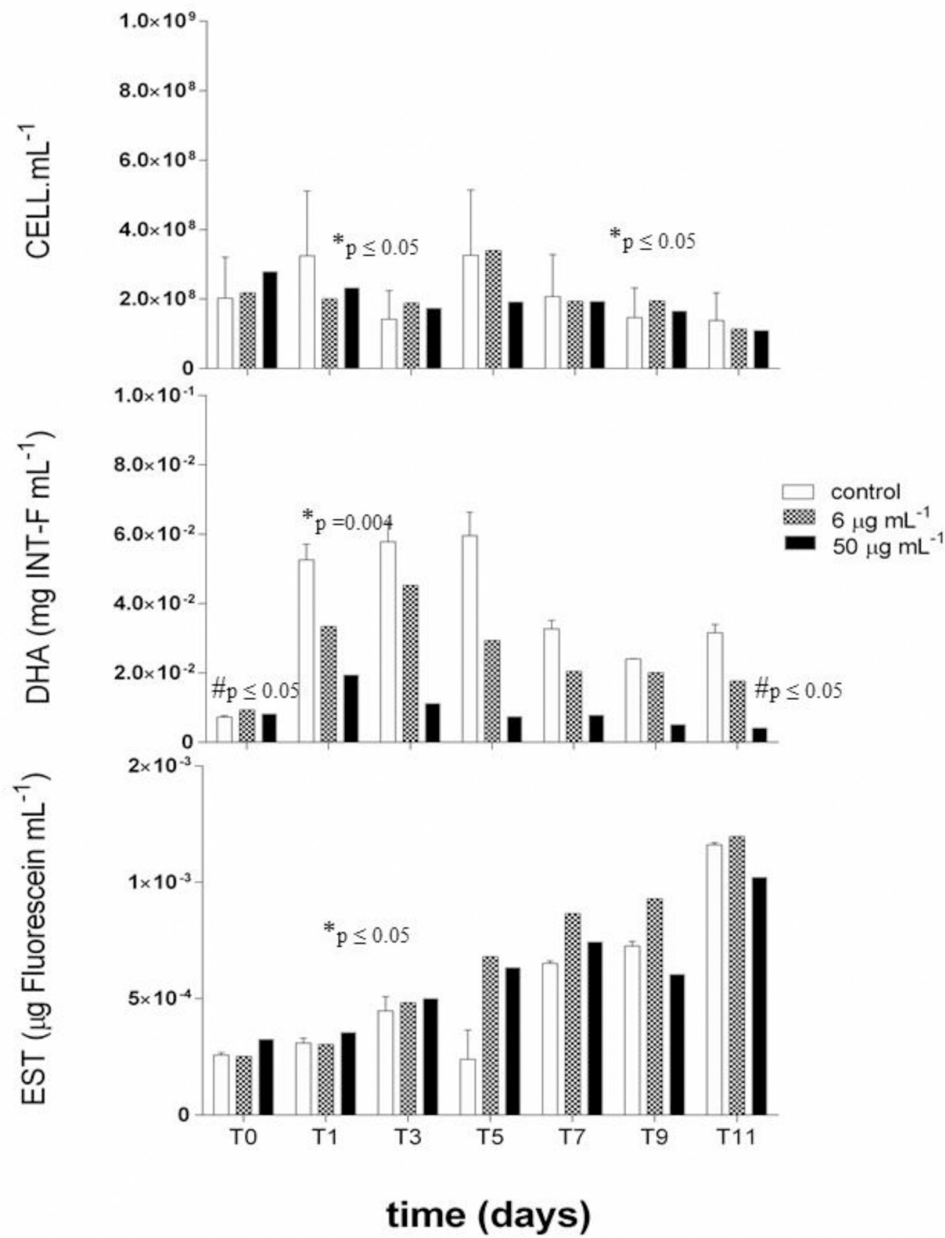
Number of cells (cell.mL^-1^), and dehydrogenase (DHA) (mg INT-F.mL^-1^) and esterase (EST) (μg Fluorescein.mL^-1^) activities in the presence of copper (Cu) at concentrations of 0 (control), 6 and 50 μg.mL^-1^. Significant differences in exposure time (*time) and absence / presence of metal (#concentration).

The bacterial consortia were dominated by the *Pseudomonas stutzeri* W228 strain, due sequences showing only this strain (degree of similarity 73–62%). The number of bacterial cells in the presence of Cu was similar to control with 10^8^ cells.mL^-1^ (Fig 2). There was a significant difference (p ≤ 0.05) in the number of cells throughout the experiment: from Time 3 (T3) to Time 5 (T5), when the number of cells increased; and from T5 to T11 when there was a decrease.

Dehydrogenase activity (DHA) is affected by Cu concentration (p ≤ 0.05) and exposition time to this metal (p = 0.004). A significant difference between the control (T0) and 50 μg.mL^-1^ of Cu (T11), and DHA activity showed a 20% reduction compared to 6 and 50 μg.mL^-1^(T11). Throughout the experiment, DHA activity showed a significant increase between T0 and T3 (Fig 2).

The esterase (EST) enzyme activity tended to increase from T0 in all Cu’s treatments, reaching maximum values in T11, with the highest values recorded for the concentration of 6 μg.mL^-1^ of Cu (Fig 2). It is worth mentioning that the lowest esterase activity was registered for Cu at the concentration of 50 μg.mL^-1^. The esterase activity showed a significant difference from T0 to T11 (p ≤ 0.05) (Fig 2). The significance level presented p ≤ 0.05 and the results are shown in Table 1.

**Table 1:** Kruskal-Wallis summary (P-values) of the parameters analyzed during bioassays with *P*. *stutzeri* exposed to metals.

Metals	Factors	Cells	DHA	EST
Cu	Concentration	0.961	**≤ 0.05**	0.314
	Time	**≤ 0.05**	**0.004**	**≤ 0.05**
Pb	Concentration	**0.040**	0.316	0.751
	Time	**0.011**	**≤ 0.05**	**≤ 0.05**
Cu + Pb	Concentration	0.607	0.256	0.923
	Time	**0.001**	**≤ 0.05**	**≤ 0.05**

Significant effects are indicated in bold. Cells: Number of Cells; DHA: Dehydrogenase activity; EST: Esterase activity.

**Fig 3:**
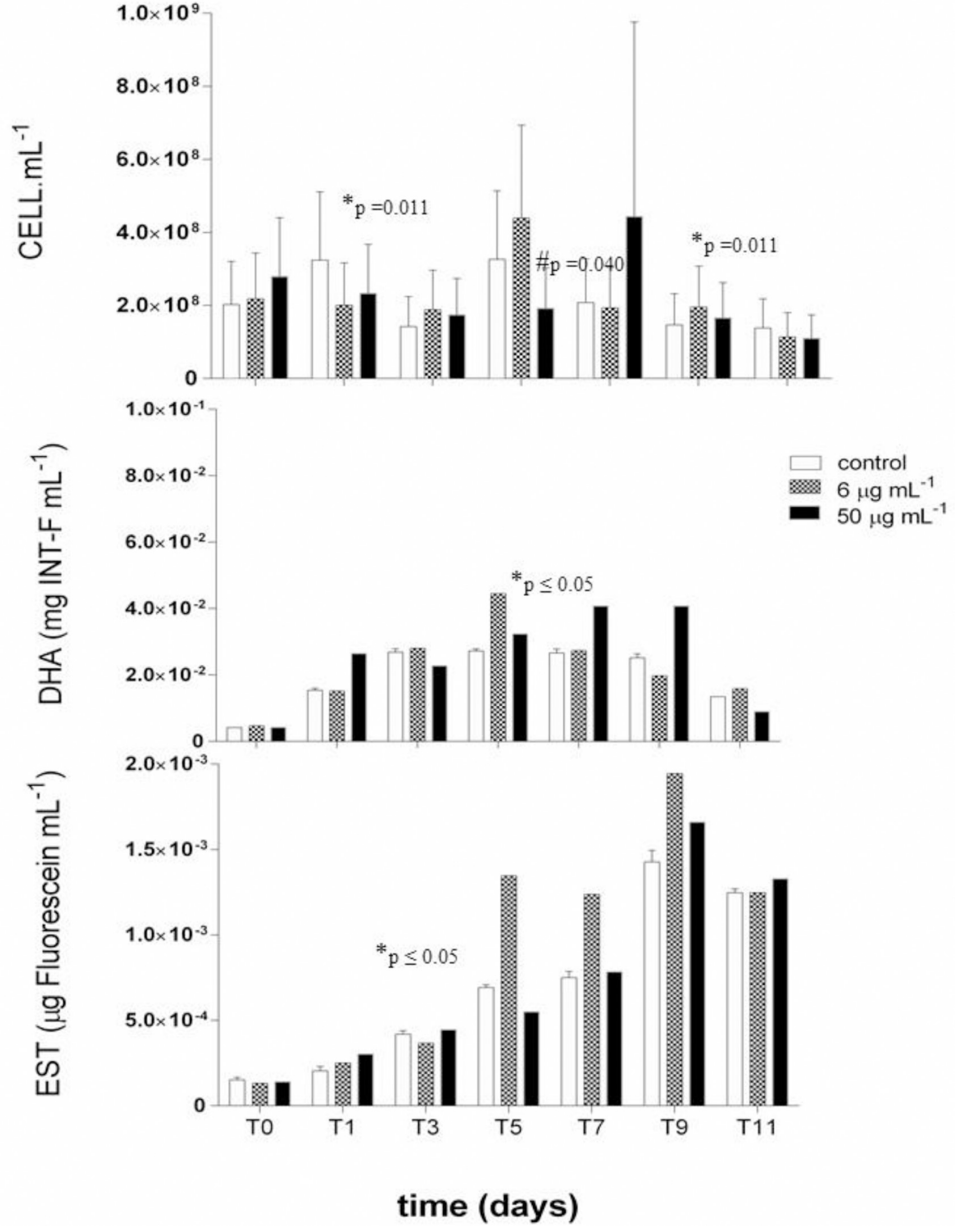
Number of cells (cell.mL^-1^), and dehydrogenase (DHA) (mg INT-F.mL^-1^) and esterase (EST) (μg Fluorescein.mL^-1^) activities in the presence of lead (Pb) at concentrations of 0 (control), 6 and 50 μg.mL^-1^. Significant differences in exposure time (*time) and absence / presence of metal (#concentration).

The number of cells in the presence of Pb also did not exceed 10^8^ cells.mL^-1^, that is, it remained equivalent to the control treatment (Fig 3). The largest biomass of 5.4 x 10^8^ cells.mL^-1^ was found in the concentration of 6 μg.mL^-1^ of Pb in T5. The statistical analysis showed that only this parameter presented significant difference in the variable time (p = 0.011) x concentration (p = 0.040). It is possible to observe a significant difference in the number of cells between concentrations 6 and 50 μg.mL^-1^ and between T0, T5, and T11, indicating that time and exposure influenced cell growth (Table 1). We observed a more extensive biofilm formation in the presence of Pb, which may have immobilized this metal to the extracellular polymeric substances (EPS) of biofilm, probably explaining the maintenance of cell biomass in the presence of Pb.

The lowest DHA in the presence of Pb was recorded at a concentration of 50 μg.mL^-1^ in T0 (4.18 x 10^−3^ μg INT-F.mL^-1^) (Fig 3). The DHA increased from T0 to T9, showing significance (p ≤ 0.05) over the time of the experiment (Table 1). The highest value was reached at T5 (4.40 x 10^−2^ μg INT-F.mL^-1^) at the Pb concentration of 6 μg.mL^-1^. Esterase activity increased throughout the bioassay for all concentrations tested (Fig 3). The lowest activity of esterase was observed in T0, while the maximum activity occurred in T9 (1.94 x 10^−3^ μg of fluorescein.mL^-1^), both linked to the treatment with 6 μg.mL^-1^ of Pb, showing significant difference over the time of the experiment (p ≤ 0.05). It is worth mentioning that the parameters DHA and EST showed a significant difference only for the time variable of exposure to Pb (Table 1).

**Fig 4:**
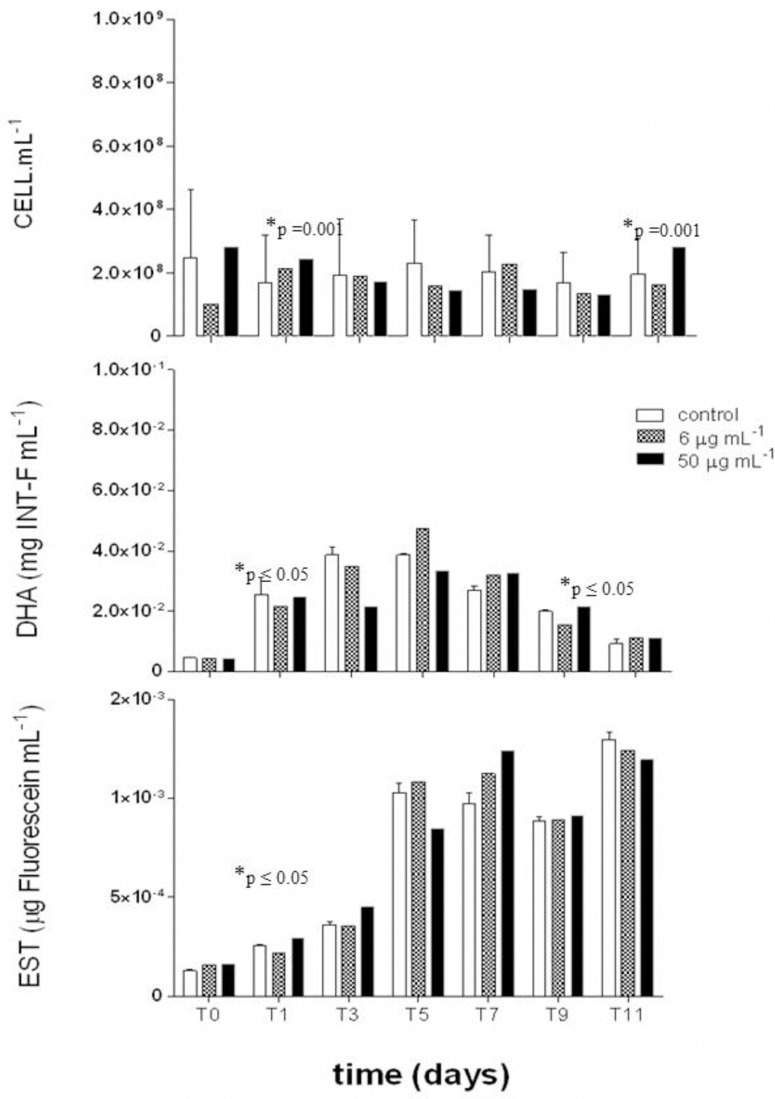
Number of cells (cell.mL^-1^), and dehydrogenase (DHA) (mg INT-F.mL^-1^) and esterase (EST) (μg Fluorescein.mL^-1^) activities in the presence of copper+lead (Cu+Pb) at concentrations of 0 (control), 6 and 50 μg.mL^-1^. Significant differences in exposure time (*time) and absence / presence of metal (#concentration).

For the bioassay in which the culture medium was enriched with Cu and Pb, in equal proportions (Fig 4), it was observed a little variation in the number of cells again, remaining at 10^8^, with the highest number recording 5,03 x 10^8^ cells.mL^-1^ in 50 μg.mL^-1^ in T0. This microbiological parameter showed a significant difference during the experiment (p = 0.001), in the concentration of 50 μg.mL^-1^, there was a decline in the number of cells between T0 and T9, with an increase in T11 (Table 1).

DHA was lower at T0 for all treatments (Fig 4), but increased to T5 when maximum activity was observed for all tested concentrations (mean: 4.08 x 10^−2^ μg of INT-F.mL^-1^). Subsequently, DHA activity decreased until the end of our bioassay period. DHA activity showed significant difference over time (p ≤ 0.05), presenting a significant increase between T0 and T5 and a decline between T7 and T11 (Table 1). The esterase activity increased during the 11-day bioassay for all tested Cu + Pb concentrations. The lowest values were observed in T0 (1.62 x 10^−4^ μg of fluorescein.mL^-1^) and the highest values were recorded in T11 (1.30 x 10^−3^ μg of fluorescein.mL^-1^), both in control (Fig 4). The activity of esterase showed a significant increase between the times T0 and T11 (p ≤ 0.05) (Table 1).

**Fig 5:**
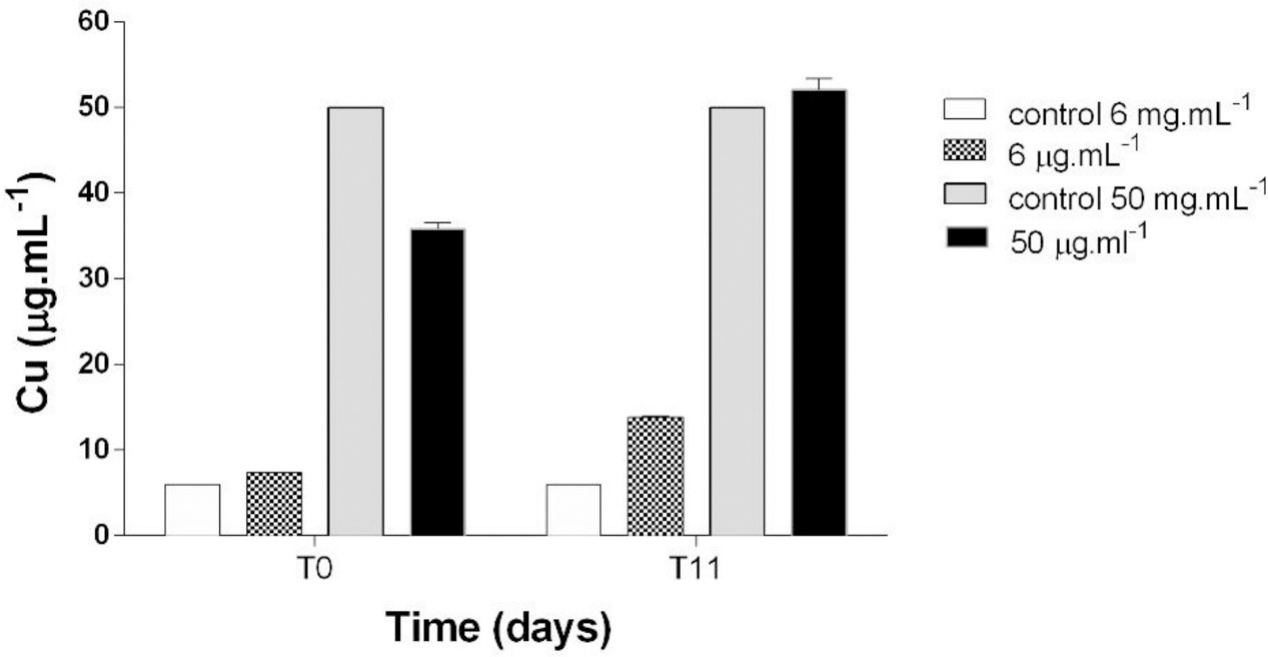
Quantification of Copper (Cu) at T0 and T11 in filtered culture media at concentrations of 6 and 50 μg.mL^-1^.

**Fig 6:**
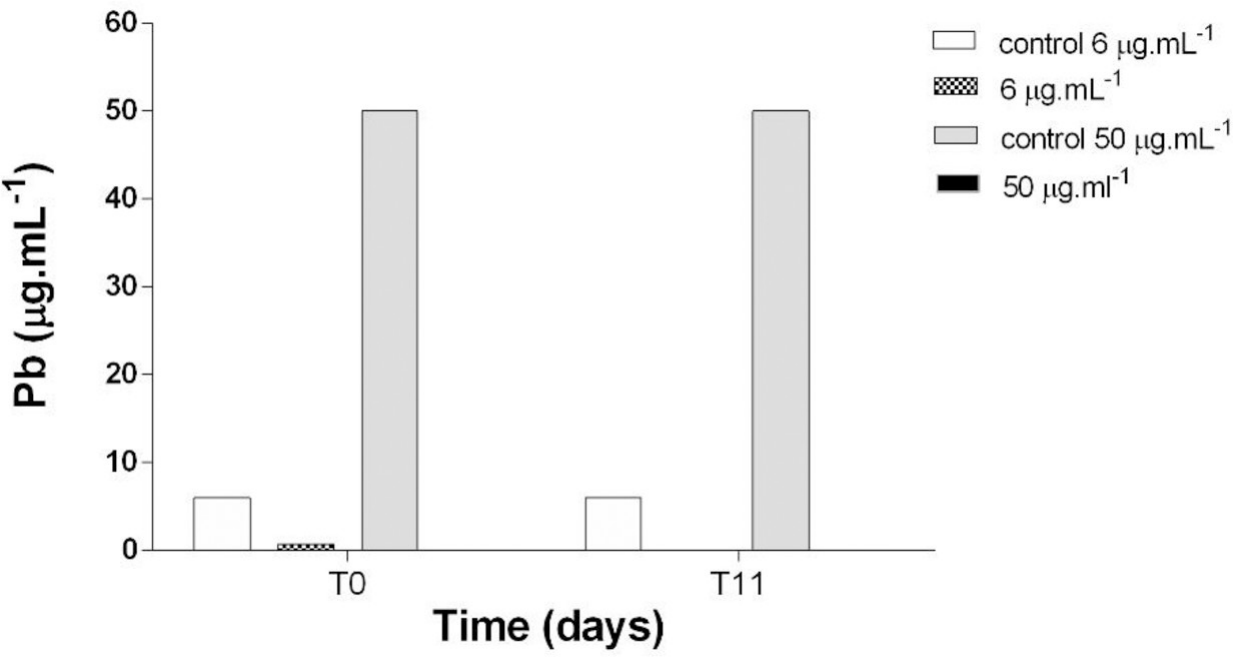
Quantification of Lead (Pb) at T0 and T11 in filtered culture media at concentrations of 6 and 50 μg.mL^-1^.

**Fig 7:**
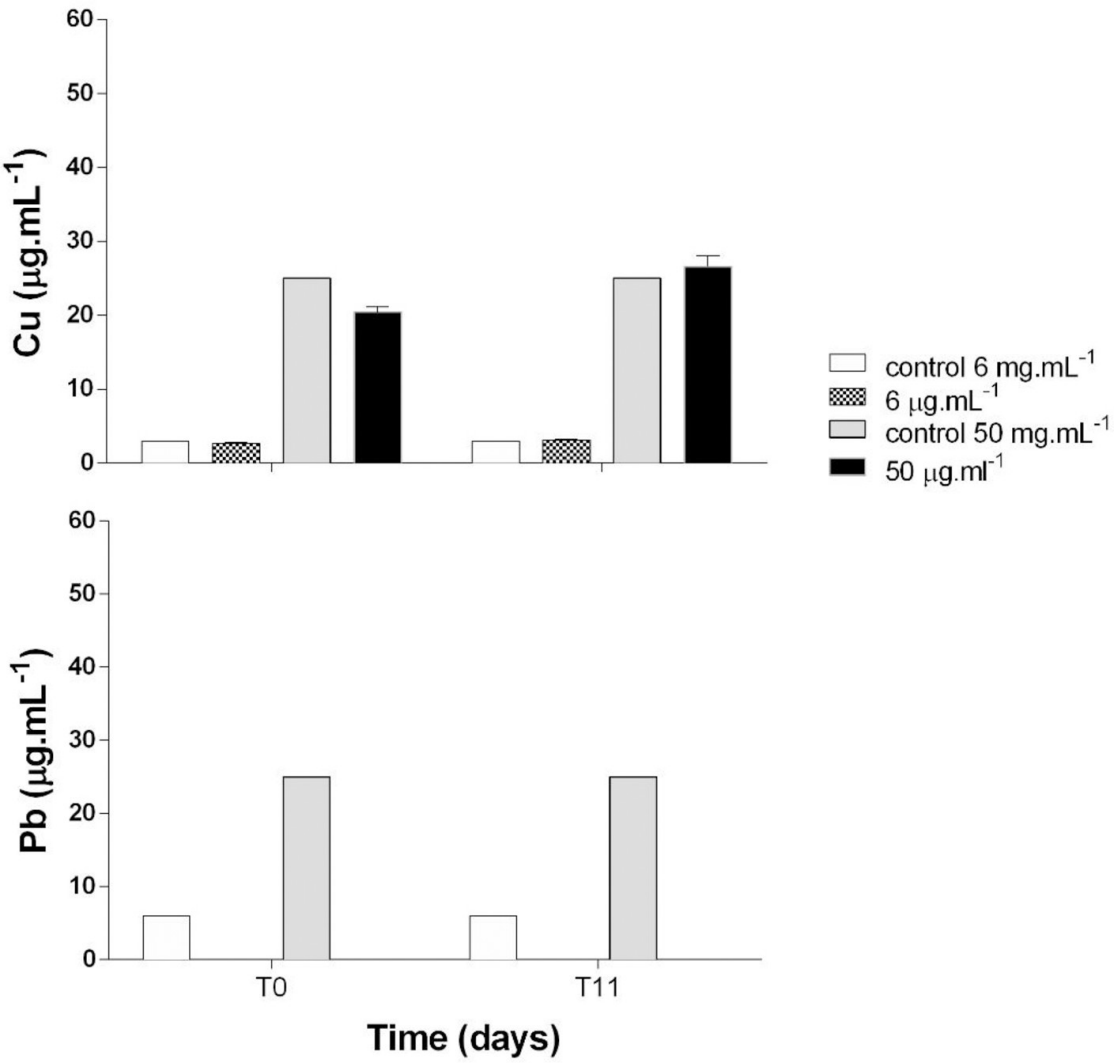
Quantification of Copper (Cu) and Lead (Pb) at T0 and T11 in filtered culture medium at concentrations of 6 and 50 μg.mL^-1^.

Figs 5, 6 and 7 present individual and combined Cu and Pb quantifications in filtered culture media at T0 and T11 from the bioassay to assess their behavior. Cu showed lower concentrations at T0 compared to T11, indicating desorption of this element (Fig 5). No Pb was detected in the filtered medium at any of the concentrations, a fact corroborated by the results found in the control sample (Fig 6). Quantitation of Cu + Pb in the filtered medium showed that Cu again exhibited desorption and Pb concentrations were below detection levels (Fig 7).

In order to evaluate the control samples and the treatments at 50 μg.mL^-1^ concentration together, a PCA analysis was performed, using all parameters (number of cells—Cells; dehydrogenases—DHA; esterase—EST, and presence/absence of metal).

**Fig 8:**
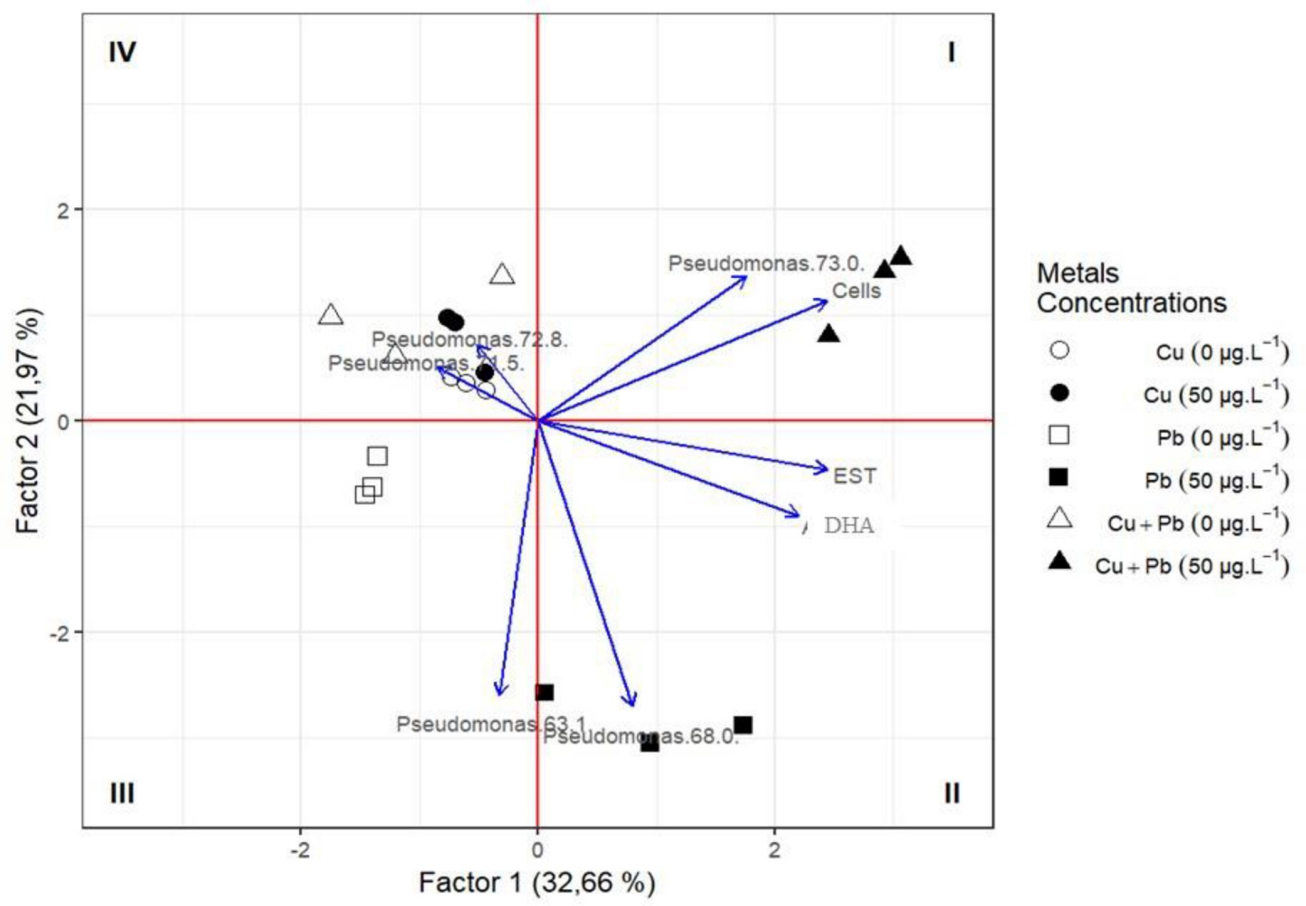
Multivariate principal component analysis (PCA) of *Pseudomonas stutzeri* in presence/absence (0 and 50 μg.mL^-1^) and exposed to different metals (Cu, Pb, Cu + Pb).

The PCA showed that the sum of the two main factors represented 54.63% of the variation in the results. Factor 1 represented 32.66% of the variation and showed a positive correlation with the activity of dehydrogenase (DHA) and esterase (EST) and the number of bacterial cells (Cells). Factor 2 represented 21.97% of the data variability but did not correlate positively with factor 1. The strains *Pseudomonas* 63.1% and 68% were negatively correlated with factor 2 (Fig 8).

The spacing of 3 sets of data is observed, which are related to the final concentration of 50 μg.mL^-1^ of Cu + Pb (quadrant I), Pb (quadrant II) and of Cu (quadrant IV) that are also correlated with the control Cu and Cu + Pb.

They distanced themselves due not only to the gene expression of *P*. *stutzeri*, but also due to the results of DHA and EST, which were responsible for cell maintenance. It is possible to infer that Cu was desorbed and Pb, due to its toxicity, was removed by EPS, which is a process governed by Pb, in the case of the combined bioassay (Cu + Pb), where *P*. *stutzeri* can be considered resistant to Pb (Fig 8).

## 4. Discussion

The only observed bacterial strain in the bacterial consortia was *Pseudomonas stutzeri* W228 **Lehman and Neumann, 1896–1927**. It is considered cosmopolitan, with great physiological and biochemical diversity such as denitrification, nitrogen fixation and degradation of polyaromatics [10, 37], and metallic biosorption [38–41]. The species has a wide range of growth temperatures, ranging from 4°C to 45°C, with ideal temperature of growth is approximately 35°C. These strains do not grow at pH less than 5, a factor that probably helped in the selection of this species in bioassays, since the media were buffered [42]. Another important feature is that they are facultative anaerobic in the presence of nitrate [43].

The number of cells, the activities of dehydrogenase and esterases, as well as the quantification of metals in the filtrate of the culture medium after growth, allowed us a better understanding of the metabolic behavior of *P*. *stutzeri* W228 in the presence of Cu and Pb alone or in combination.

Several studies have used dehydrogenase activity as a marker of toxicity of petroleum hydrocarbons, pesticides, and metals to soil and sediment bacteria [5, 44–51]. In our study, the parameters of esterases and dehydrogenases activities demonstrated, respectively, a pattern for the hydrolysis of biopolymers and intracellular supply with compounds <600 Da [32] for energy production, sufficient to maintain the number of cells.

Chequer et al. [44] observed the same pattern in the esterases and dehydrogenases enzyme activities on impacted soil by diesel oil, maintaining the number of cells in sediments. The same activity pattern was also observed in sediments from Guanabara Bay, Brazil, due to contamination by Cu, Zn and Pb [45, 52]. In environments contaminated by stressors such as metals, the increased activity of esterases, extracellular enzymes linked to the hydrolysis of organic matter [31], indicates that microorganisms are capable of completing their life cycle [53].

The non-biodegradable and recalcitrant nature of heavy metals guarantees its prolonged presence in the environment and its high aquatic solubility promotes bioaccumulation and biomagnifications in the trophic web [54]. Superficial sediments of Sepetiba Bay have concentrations of Pb and Cu ranging from undetectable levels to 54.0 mg.kg^-1^ and from 1.64 to 16 mg.kg^-1^, respectively [16, 55].

Our results with *P*. *stutzeri* W228 showed biosorption of Pb by the EPS. Mattuschka et al. [41] found similar results of biosorption with *P*. *stutzeri* AG 259 for Pb instead of Cu, following the order of: Ag> Pb> Cu> Zn. Joo et al. [40] concluded that the species is a promising biosorbent since lyophilized *P*. *stutzeri* cells have a higher biosorption affinity for Pb than Cd.

The abilities of EPS production and biosorption of metals and multimetals by *P*. *stutzeri* were evaluated by Maalej et al. [38], showing hierarchy in the biosorption capacity of Pb >> Co> Fe> >> Cd. Badar et al. [39] showed an efficiency of 90% of Cu removal by *P*. *stutzeri*, when isolated from contaminated soil and maintained in bioreactors in the presence of Cu. Sunilkumar et al. [56] isolated 25 species of bacteria tolerant to Pb, Cr, Cd and Hg and among them was *P*. *stutzeri*. Our results corroborated with Waite et al. [5] when it was observed the Pb biosorption and the Cu efflux by bacterial pools in aqueous solutions. Also, several strains of *P*. *stutzeri* have been described as having a high biosorption capacity by EPS for Cr, Co, Mn, Ti, U, V, Zn [57], Pb [41] and Ni [58].

Bacterial cells invest in the production of EPS due to the biosorption of Pb and also in the production of proteins capable of effacing Cu [5, 39, 59, 60]. Cu is a micronutrient that is a constituent of several enzymes [61]. However, in high concentrations, it becomes toxic, interacting with thiol groups and destabilizing iron-sulfur cofactors, thus competing with other metals for binding sites of proteins [19]. *P*. *stutzeri* strains, when exposed to concentrations that exceed the cellular consumption of Cu as a micronutrient, induced the desorption, probably linked to the CusC (F) BA Cu / Ag efflux pump [61–63].

Facilitation of the Pb biosorption process by *P*. *stutzeri* W228 is linked to the production of EPS [59, 60, 64], which has functional groups such as carboxyl (COOH), phosphate (R- HPO4), sulfhydryl (R-SH), amine (R-NH2), phenolic (R-C6H5OH) and hydroxyl (R-OH) [65–67]. Besides, *Pseudomonas* species have various adhesins that act on the initial binding to a substrate, facilitating the formation of the biofilm [42].

Based on the positioning of our samples in the PCA, it is possible to infer that *P*. *stutzeri* W228 was able to metabolically provide, through the activity of dehydrogenases and esterases, subsidies to maintain the number of cells and consequently cause the efflux of Cu and remove Pb due to EPS formation. This behavior pattern of bacterial metabolic activity in contaminated environments was reported by other authors [5, 39, 45, 52, 60]. Together, these findings indicate that, in the presence of multimetals, *P*. *stutzeri* presents preferential biosorption of Pb, with the inertization of more toxic metals (Pb) over less toxic metals (Cu).

High concentrations of metals alter bacterial diversity, but, in the long-term, promote the development of resistant or tolerant species in the environment [68], also indicating that metals are bioavailable in the environment [5, 29, 52, 60, 69]. Thus, it was possible to select *P*. *stutzeri* W228 resistant to Cu and Pb, with Pb's biosorbent capacity, from surface sediments samples contaminated by multimetals.

## 5. Conclusions

Strains of *P*. *stutzeri* W228 are candidates to be used in bioremediation technology in environments impacted by metals, since they preferentially remove Pb. The enzymes dehydrogenases and esterases are biomarkers that can be used to indicate the maintenance of the number of cells, when bacteria are exposed to metals and multimetals.

## References

[1] Gupta A., Joia J., 2016 Microbes as Potential Tool for Remediation of Heavy Metals: A Review. J. Microb. Biochem. Technol. 8, 364–372. 10.4172/1948-5948.1000310.

[2] Niemeyer J. C., Moreira-Santos M., Ribeiro R., Rutgers M., Nogueira M. A., da Silva E. M., et al 2015 Ecological Risk Assessment of a Metal-Contaminated Area in the Tropics. Tier II: Detailed Assessment. PloS One,10(11), e0141772 10.1371/journal.pone.0141772 26528915 PMC4631348

[3] Caicedo Pineda G.A., Márquez Godoy M.A., 2019 Effect of *Acidithiobacillus* thiooxidans-Cysteine Interactions on Pyrite Biooxidation by Acidithio bacillus ferrooxidans in the Presence of Coal Compounds. Brazilian J. Chem. Eng. 36, 681–692. 10.1590/0104-6632.20190362s20180294

[4] Işıldar A., van Hullebusch E.D., Lenz M., Du Laing G., Marra A., Cesaro A., et al, 2019 Biotechnological strategies for the recovery of valuable and critical raw materials from waste electrical and electronic equipment (WEEE)–A review. J. Hazard. Mater. 362, 467–481. 10.1016/j.jhazmat.2018.08.050. 30268020

[5] Waite C.C. da C, da Silva G.O.A., Bitencourt J.A.P., Sabadini-Santos E., Crapez M.A.C., 2016 Copper and lead removal from aqueous solutions by bacterial consortia acting as biosorbents. Mar. Pollut. Bull. 109, 386–392. 10.1016/j.marpolbul.2016.05.044 27236233

[6] Ayangbenro A.S., Babalola O.O., 2017 A new strategy for heavy metal polluted environments: A review of microbial biosorbents. Int. J. Environ. Res. Public Health 14 10.3390/ijerph14010094. PMC5295344 28106848

[7] Wuana R.A., Okieimen F.E., 2011 Heavy Metals in Contaminated Soils: A Review of Sources, Chemistry, Risks and Best Available Strategies for Remediation. ISRN Ecol., 1–20. 10.5402/2011/402647.

[8] Yazdankhah S., Skjerve E., Wasteson Y., 2018 Antimicrobial resistance due to the content of potentially toxic metals in soil and fertilizing products, in: Microbial Ecology in Health and Disease. Taylor & Francis, p. 1548248 10.1080/16512235.2018.1548248 PMC7273308 32547355

[9] Jarosławiecka A., Piotrowska-Seget Z., 2014 Lead resistance in micro-organisms. Microbiol. (United Kingdom) 160, 12–25. 10.1099/mic.0.070284-0. 24124204

[10] Lalucat J., Bennasar A., Bosch R. Garcıa-Valde´s E., Palleroni N. J. 2006 Biology of Pseudomonas stutzeri. Microbiology and Molecular Biology Reviews, 6 2006, p. 510–547 Vol. 70, No. 2 1092-2172/06/$08.000 10.1128/MMBR.00047-05 16760312 PMC1489536

[11] Niti C, Sunita S, Kamlesh K, Rakesh K 2013 Bioremediation: An emerging technology for remediation of pesticides. Res J Chem Environ 17:88–105.

[12] Das S., Dash H. R. and Chakraborty J. 2016 Genetic basis and importance of metal resistant genes in bacteria for bioremediation of contaminated environments with toxic metal pollutants. Appl Microbiol Biotechnol.100:2967–2984. 26860944 10.1007/s00253-016-7364-4

[13] Chandrangsu Pete, Rensing Christopher, and Helmann John D. 2017 “Metal Homeostasis and Resistance in Bacteria.” Nature Reviews Microbiology 15(6): 338–50. 10.1038/nrmicro.2017.15 28344348 PMC5963929

[14] Paraquetti H. H. M.; Ayres G. A.; Almeida M.; Molisani M. M. and Lacerda L. D. 2004 Mercury, distribution speciation and flux in the Sepetiba Bay tributaries. Water Res., 38: 1439–1448. 15016520 10.1016/j.watres.2003.11.039

[15] Fonseca E. M.; Baptista-Neto J. A. and Silva C. G. 2013 Heavy metal accumulation in mangrove sediments surrounding a large waste reservoir of a local metallurgical plant, Sepetiba Bay, SE, Brazil. Environ Earth Sci., 70: 643–650.

[16] Pinto A.F.S., Ramalho J.C.M., Borghi L., Carelli T.G., Plantz J.B., Pereira E., et al, 2019 Background concentrations of chemical elements in Sepetiba Bay (SE Brazil). Journal of Sedimentary Environments, 4 (1): 108–123.

[17] Baptista-Neto J.A, Smith B.J, McAlister J.J. 2000 Heavy metal concentrations in surface sediments in a nearshore environment, Jurujuba Sound, SE Brazil. Environ Pollution 109(1):1–9. 10.1016/s0269-7491(99)00233-x 15092907

[18] Madigan M.T., Martinko J.M., Parker J., Fernández M.G., Pérez M.S., 2003 Biología de los microorganismos: Brock. Prentice Hall, Madrid.

[19] Rademacher C.; Masepohl B. 2012 Copper-responsive gene regulation in bactéria. Microbiology. 158, 2451–2464. 10.1099/mic.0.058487–0 22918892

[20] Brasil, 2016 Departamento de Ações Programáticas Estratégicas Atenção à saúde dos trabalhadores expostos ao chumbo metálico. Editora do Ministério da Saúde, Brasília.

[21] Abuchacra P.F.F., Aguiar V.M.C., Abuchacra R.C., Baptista Neto J.A., Oliveira A.S., 2015 Assessment of bioavailability and potential toxicity of Cu, Zn and Pb, a case study in Jurujuba Sound, Rio de Janeiro, Brazil. 100, 414–425. 10.1016/j.marpolbul.2015.08.012 26320979

[22] Brons J.K. & Van Elsas J. D. 2008 Analysis of bacterial communities in soil by use of denaturing gradient gel electrophoresis and clone libraries, as influenced by different reverse primers. Appl Environ Microbiol 74:2717–2727. 18310425 10.1128/AEM.02195-07 PMC2394888

[23] Heuer H. and Smalla K. 1997 Application of denaturing gradient gel electrophoresis and temperature gradient gel electrophoresis for studying soil microbial communities In: van Elsas JD, Wellington EMH & Trevors J (eds) Modern Soil Microbiology. Marcel Dekker Inc, New York 353–373.

[24] Heuer H., Wieland J., Schönfeld J., Schönwälder A., Gomes N. C. M. and Smalla K. 2001 Bacterial community profiling using DGGE or TGGE analysis In: Rouchelle PA (ed) Environmental Molecular Microbiology: Protocols and Applications. Horizon Scientific Press Wymondham, UK, 629:177–190.

[25] Sambrook J & Russel DW. 2001 Molecular Cloning (3rd). Cold Spring Harbour Laboratory Press, Cold Spring Harbour, New York.

[26] Green M. R. and Sambrook J. 2012 Molecular Cloning (4th). Cold Spring Harbour Laboratory Press, 633 Cold Spring Harbour, New York.

[27] Bloem J., Bar-Gilissen M.J., Cappenberg T.E., 1986 Fixation, Counting, and Manipulation of Heterotrophic Nanoflagellates. Appl Environ Microbiol. 12; 52(6): 1266–1272. 16347232 10.1128/aem.52.6.1266-1272.1986 PMC239220

[28] Kepner R. L., Pratt J. R., 1994 Use of fluorochromes for direct enumeration of total bacteria in environmental samples: past and present. Microbiol. Rev. 58:603–615. 7854248 10.1128/mr.58.4.603-615.1994 PMC372983

[29] Pennafirme S., Lima I., Bitencourt J. A., Crapez M. A. C., Lopes R. T. 2015 Microbial biofilm study by synchrotron X-ray microscopy. Radiation Physics and Chemistry 116: 116–119.

[30] Kenarova A., Radeva G., Traykov I., Boteva S., 2014. Community Level Physiological Profiles of Bacterial Communities Inhabiting Uranium Mining Impacted Sites. Ecotoxicol Environ Saf. 100:226–232. 10.1016/j.ecoenv.2013.11.012 24315773

[31] Stubberfield L. C. F., Shaw P.J.A., 1990 A comparison of tetrazolium reduction and FDA hydrolysis with other measures of microbial activity. J. Microbiol. Methods 12, 151–162.

[32] Weiss M., Abele U., Weckesser J., Welte W., Schiltz E., Schulz G., 1991 Molecular Architecture and Electrostatic Properties of a Bacterial Porin. Science. 254 pp. 1627–1630. 1721242 10.1126/science.1721242

[33] R Core Team; 2015 R: A language and environment for statistical computing. R Foundation for Statistical Computing, Vienna, Austria URL https://www.R-project.org/.

[34] Oksanen J., Blanchet F.G., Kindt R., Legendre P., Minchin P.R., O’Hara R.B., et al, 2013 Vegan: Community Ecology Package. R package version 2.0–10.

[35] Milligan G.W., Cooper M.C. 1985 An examination of procedures for determining the number of clusters in a data set. Psychometrika 50, 159–179. 10.1007/BF02294245

[36] Field A. 2009 Discovering Statistics Using SPSS. 3rd Edition, Sage Publications Ltd, London.

[37] Parthipan P; Elumalai P; Machuca L; Rahman P; Murugan K; Rajasekar A. 2017. Biosurfactant and Degradative Enzymes Mediated Crude Oil Degradation by Bacterium *Bacillus subtilis* A1. Frontiers in Microbiology. 8 10.3389/fmicb.2017.00193 28232826 PMC5299021

[38] Maalej H, Hmidet N, Boisset C, Buon L, Heyraud A and Nasri M 2014 Optimization of exopolysaccharide production from *Pseudomonas stutzeri* AS22 and examination of its metal-binding abilities. Journal of Applied Microbiology ISSN 1364-5072. 10.1111/jam.12688 25376444

[39] Badar U, Shoeb E, Qureshi F. M., Akhtar J., Ahmed N. 2013 Removal of Copper via bioreactor by soil isolate *Pseudomonas stutzeri*. Academic Research International. ISSN-L: 2223–9553, ISSN: 2223-9944 Vol. 4 No. 3.

[40] Joo, J. H., Oh, S. E., Hassan, H. A., Son, J.S. and Lim, K. S. 2009. Biosorptive Capacity of Pb(II), Cd(II) and Cu(II) by Lyophilized Cells of Pseudomonas stutzeri. Annals of the 9th International Conference of the East and Southeast Asia Federation of Soils Science Societies. p. 450–451.

[41] Mattuschka B., Straube G., Trevors J. T. 1994 Silver, copper, lead and zinc accumulation by *Pseudomonas stutzeri* AG259 and *Streptomyces albus*: electron microscopy and energy dispersive X-ray studies. BioMetals 7:201–208.

[42] Rosselló-Mora R, Amann R. 2001. The species concept for prokaryotes. FEMS Microbiol. Rev. 2001;25(1):39‐67. 10.1111/j.1574-6976.tb00571.x 11152940

[43] Härtig E, Schiek U, Vollack KU, Zumft WG. 1999 Nitrate and nitrite control of respiratory nitrate reduction in denitrifying Pseudomonas stutzeri by a two-component regulatory system homologous to NarXL of Escherichia coli.J Bacterio.l.;181(12):3658‐3665. 10.1128/jb.181.12.3658-3665.1999 PMC93841 10368138

[44] Chequer L. P. T.; Bitencourt J. A. P.; Waite C. C. C.; Sabadini-Santos E.; Franco D. C.; Alves J. R. et al, 2017 Response of mangrove propagules to the presence of oil and hydrocarbon degrading bacteria during an experimental oil spill. Latin American Journal of Aquatic Research, v. 45, p. 814–821.

[45] Fonseca E. M., Baptista Neto J. A., Crapez M. C., McAlister J. J., Fernandez M. A. and Bispo M. G. 2009 Bioavailability of heavy metals in Guanabara Bay, Rio de Janeiro, (Brazil). J Coastal Res 56:802–806.

[46] Shen G., Lu Y., Zhou Q., Hong J. 2005 Interaction of polycyclic aromatic hydrocarbons and heavy metals on soil enzymes. Chemosphere. 61(8):1175–82. 16263387 10.1016/j.chemosphere.2005.02.074

[47] Mora A. P., Ortega-Calvo J. J., Cabrera F., Madejón E. 2005 Changes in enzyme activities and microbial biomass alter “in situ” remediation of a heavy metal contaminated soil. Appl. Soil Ecol., v. 28, p125–137.

[48] Kizilkaya R., Aaskin T., Bayrakli B., Saglam M. 2004 Microbiological characteristics of soils contaminated with heavy metals. Europ. J. Soil Biol. 40:95–102.

[49] Baptista-Neto J. A.; Crapez M.; McAlister J. J., and Vilela C.G. 2004 Concentration and bioavailability of heavy metals in sediments from Niterói Harbour (Guanabara Bay/S.E. Brazil). Journal of Coastal Research, v. 20, p. 749–208.

[50] Crapez M. A. C., Baptista Neto J. A., Bispo M. G. S. 2003 Bacterial Enzymatic Activity and Bioavailability of Heavy Metals in Sediments From Boa Viagem Beach, Guanabara Bay, RJ, Brazil. Anuário do Instituto de Geociências, UFRJ, v.26 p.60–68.

[51] Irha N., Slet J. and Petersell V. 2003 Effect of heavy metals and PAH on soil accessed via dehydrogenase assay. Environ. Intern., 28:779–782. 10.1016/S0160-4120(02)00124-1 12605927

[52] Sabadini-Santos E., Silva T. S., Lopes-Rosa T., Mendonça-Filho J. C., Santelli R. E., Crapez M. A. C. 2014 Microbial Activities and Bioavailable Concentrations of Cu, Zn, and Pb in Sediments from a Tropic and Eutrothicated Bay. Water Air Soil Pollut 225: 1–11.

[53] Anderson T.H. and Domsch K.H. 2010 Soil microbial biomass: The eco-physiological approach. Soil Biology & Biochemistry 42, 2039–2043.

[54] Gupta Pratima, and Diwan Batul. 2017 “Bacterial Exopolysaccharide Mediated Heavy Metal Removal: A Review on Biosynthesis, Mechanism and Remediation Strategies.” Biotechnology Reports 13: 58–71. 10.1016/j.btre.2016.12.006. 28352564 PMC5361134

[55] Fonseca E. F.; Baptista-Neto J. A. & Silva C. G. 2013 Heavy metal accumulation in mangrove sediments surrounding a large waste reservoir of a local metallurgical plant, Sepetiba Bay, SE, Brazil. Environ Earth Sci., 70: 643–650.

[56] Sunilkumar C. R., Rachel L., Bhavya G., Swati K., Sridhar R., Samaga J., et al, 2017. Practiced Gram negative bacteria from dyeing industry effluents snub metal toxicity to survive. Journal of Applied Biology & Biotechnology Vol. 5 (04), pp. 037–042. 10.7324/JABB.2017.50406

[57] Hemme Christopher L., et al 2016 “Lateral Gene Transfer in a Heavy Metal-Contaminated-Groundwater Microbial Community.” mBio 7(2). 10.1128/mBio.02234-15 PMC4817265 27048805

[58] Alboghobeish Hoda, Tahmourespour Arezoo, and Doudi Monir. 2014 “The Study of Nickel Resistant Bacteria (NiRB) Isolated from Wastewaters Polluted with Different Industrial Sources.” Journal of Environmental Health Science and Engineering 12(1): 1–7. Journal of Environmental Health Science and Engineering. 24475932 10.1186/2052-336X-12-44 PMC3931474

[59] Jung A., Eisheuer S., Cserti E., Leicht O., Strobel W., Möll A., et al, 2015 Molecular toolbox for genetic manipulation of the stalked budding bacterium *Hyphomonas neptunium*. Appl Environ Microbiol 81:736–744. 10.1128/AEM.03104-14 25398860 PMC4277588

[60] Krepsky N.; Silva F. S.; Fontana L. F.; Crapez M. A. C. 2007 Alternative methodology for isolation of biosurfactant-producing bacteria. Brazilian Journal of Biology, 67: 117–124. 17505758 10.1590/s1519-69842007000100016

[61] Braymer J.J. and Giedroc D. P. 2014 Recent developments in copper and zinc homeostasis in bacterial pathogens. Curr Opin Chem Biol. 4;19:59–66. 10.1016/j.cbpa.2013.12.021 Epub 2014 Jan 22. ; PMCID: PMC4008645. 24463765 PMC4008645

[62] Pontel L.B, Scampoli N. L, Porwollik S, Checa S. K, McClelland M, Soncini F. C. 2014 Identification of a Salmonella ancillary copper detoxification mechanism by a comparative analysis of the genome-wide transcriptional response to copper and zinc excess. Microbiology. 160:1659–1669. 10.1099/mic.0.080473–0 24858080 PMC4117220

[63] Samanovic M. I, Ding C, Thiele D. J, Darwin K. H. 2012. Copper in microbial pathogenesis: meddling with the metal. Cell Host Microbe. 2 16;11(2):106–15. 10.1016/j.chom.2012.01.009 22341460 PMC3285254

[64] Kasai Y., Kishira H., Sasaki T., Syutsubo K., Watanabe K., Harayama S., 2002 Predominant growth of *Alcanivorax strains* in oil-contaminated and nutrient supplemented sea water. Environ. Microbiol. 4, 141–147. 12000314 10.1046/j.1462-2920.2002.00275.x

[65] D’Acunto B.; Esposito G.; Frunzo L.; Mattei M. R., Pirozzi F. 2015. Mathematical Modeling of Heavy Metal Biosorption in Multispecies Biofilms. American Society of Civil Engineers. 10.1061/(ASCE)EE.1943-7870.0001052

[66] Branda S. S., Vik S., Friedman L., Kolter R. 2005 Biofilms: the matrix revisited. Trends Microbiol. 13, 20–26. 15639628 10.1016/j.tim.2004.11.006

[67] Whitchurch C. B., Tolker-Neilsen T., Ragas P. C., Mattick J. S. 2002 Extracellular DNA required for bacterial biofilm formation. Science 295, 1487. 11859186 10.1126/science.295.5559.1487

[68] Harrison J. J., Ceri H., and Turner R. J. 2007 Multimetal resistance and tolerance in microbial biofilms. Nature Reviews Microbiology 5: 928–938. 17940533 10.1038/nrmicro1774

[69] Semple K. T., Doick K. J., Jones K. C., Burauel P., Craven A., Harms H., 2004 Defining bioavailability and bioaccessibility of contaminated soil and sediments is complicated. Environ. Sci. Technol. 38, 228Ae231A. 10.1021/es040548w 15260315

